# Affective Reactivity to a Single Bout of High-Intensity Interval Training in Schizophrenia: A Randomized Controlled Trial

**DOI:** 10.1093/schizbullopen/sgag004

**Published:** 2026-02-27

**Authors:** Markus Sanden, Guro Pauck Bernhardsen, Ketil Hanssen-Bauer, Anne Høye, Erik Johnsen, Gunnar Morken, Mona Nygård, John Abel Engh

**Affiliations:** Division of Mental Health and Addiction Services, Akershus University Hospital, 1478 Lørenskog, Norway; Institute of Clinical Medicine, Faculty of Medicine, University of Oslo, 0372 Oslo, Norway; Division of Mental Health and Addiction Services, Akershus University Hospital, 1478 Lørenskog, Norway; Division of Mental Health and Addiction Services, Akershus University Hospital, 1478 Lørenskog, Norway; Institute of Clinical Medicine, Faculty of Medicine, University of Oslo, 0372 Oslo, Norway; Department of Clinical Medicine, UiT - The Arctic University of Norway, 9037 Tromsø, Norway; Division of Mental Health and Substance Abuse, University Hospital of North Norway, 9038 Tromsø, Norway; Division of Psychiatry, Haukeland University Hospital, 5021 Bergen, Norway; Department of Clinical Medicine, University of Bergen, 5020 Bergen, Norway; Mohn Research Center for Psychotic Disorders, Haukeland University Hospital, 5036 Bergen, Norway; Department of Mental Health, Faculty of Medicine and Health Sciences, Norwegian University of Science and Technology (NTNU), 7491 Trondheim, Norway; Department of Psychiatry, St. Olavs Hospital, Trondheim University Hospital, 7006 Trondheim, Norway; Department of Mental Health, Faculty of Medicine and Health Sciences, Norwegian University of Science and Technology (NTNU), 7491 Trondheim, Norway; Department of Psychosis and Rehabilitation, Division of Mental Healthcare, St. Olavs University Hospital, 7040 Trondheim, Norway; Division of Mental Health and Addiction Services, Akershus University Hospital, 1478 Lørenskog, Norway; Division of Mental Health and Addiction, Vestfold Hospital Trust, 3103 Tønsberg, Norway

**Keywords:** schizophrenia, affective reactivity, positive and negative affect, high-intensity interval training (HIIT), physical activity, randomized controlled trial

## Abstract

**Background and Hypothesis:**

Affective disturbances are a core feature of schizophrenia, yet patients’ capacity to experience momentary affective shifts in real-world settings remains underexplored. Although consummatory pleasure appears preserved, studies suggest low levels of positive affect and high levels of negative affect in daily life. Physical exercise provides an ecologically valid context for probing short-term affective reactivity. This study examined whether high-intensity interval training (HIIT) elicited greater affective change than an active control condition (ACC), whether baseline symptom levels were associated with affective state, and whether baseline symptoms moderated affective reactivity.

**Study Design:**

In a randomized controlled trial, 69 outpatients with schizophrenia were randomized to a 12-week intervention involving either supervised HIIT or an ACC involving low-intensity, interactive video gaming. Affective state was assessed immediately before and after the 12th session using the Positive and Negative Affect Schedule (PANAS). Depressive (CDSS), negative symptoms (PANSS-N), and apathy (AES) were measured at baseline. Linear mixed-effects models tested Group × Time effects.

**Study Results:**

HIIT did not produce significantly greater changes in positive or negative affect than the active control. Across interventions, participants showed increases in positive affect and decreases in negative affect. Higher baseline symptom levels were associated with lower pre-session positive affect. Exploratory analyses indicated that participants with more severe negative symptoms showed greater positive affective gains following HIIT.

**Conclusions:**

A single session of structured activity, regardless of intensity, was associated with small but measurable affective improvements. These findings highlight the value of accessible activity-based interventions for enhancing affective well-being in schizophrenia.

## Introduction

Affective disturbances are a well-documented feature of schizophrenia, associated with reduced quality of life, lower social functioning, and clinical outcomes.^1–3^ Although individuals with schizophrenia show impairments in anticipating emotional events, sustaining affective states, and integrating emotional cues with context,^2^ studies find that their ability to experience pleasurable affective states is often intact.^4–6^ Interestingly, this preserved capacity for consummatory pleasure appears at odds with real-world reports. When affect is assessed in daily life, individuals with schizophrenia generally report lower positive affect and higher negative affect than control subjects.^7–9^ These findings may suggest difficulty in accessing affective states in everyday contexts, rather than a global affective disturbance.

High-intensity interval training (HIIT) provides a suitable context for investigating shifts in affective states in ecologically valid settings. In schizophrenia, HIIT has been associated with improvements in symptoms, cognition, and cardiometabolic health.^10–13^ However, dropout remains a challenge for the overall effect of physical activity interventions.^14^^,^^15^ A recent meta-analysis highlights the need to better understand affective responses to exercise in this population,^16^ suggesting that patients’ affective experiences during and after exercise may influence intervention outcomes. Supporting this idea, a single session of aerobic exercise has been shown to increase positive affect in depression,^17^^,^^18^ and greater post-session positive affect has been shown to predict stronger treatment response over time.^18^ Similarly, in schizophrenia, a single session of high-intensity training has been shown to increase positive affect,^19^ and brief moderate-intensity walking has also been associated with increased pleasant affect.^20^ In non-clinical populations, HIIT typically elicits reduced pleasure during exertion, followed by a post-exercise rebound in positive affect,^21^^,^^22^ though evidence on its psychological effects remains inconclusive.

Despite the clinical relevance of affective disturbances in schizophrenia,^1^^,^^23^ most research has focused on trait-level constructs such as anhedonia or negative symptoms. While valuable, these measures offer limited insight into how individuals respond emotionally to specific events.^23^ Accounting for co-occurring symptom domains that may shape affective state and reactivity could represent a more nuanced understanding. For example, apathy, depressive symptoms, and negative symptoms may bias individuals toward low-activation, low-pleasure affective states,^24^ potentially dampening both baseline affective state and reactivity to affectively salient experiences. Yet, it remains unclear to what extent these symptom profiles explain shifts in affective states.

The present study examined affective reactivity to a single session of HIIT versus an active control condition (ACC) in individuals with schizophrenia. Because affective responses to exercise may be influenced by both intensity and situational context,^21^^,^^22^ the ACC provided a structured and supervised comparison condition that differed primarily in physical intensity. We hypothesized that (1) participants in the HIIT group would show significantly greater increases in positive affect and greater decreases in negative affect from pre- to post-session compared to control subjects; (2) higher baseline levels of apathy, depressive symptoms, and negative symptoms would be associated with lower levels of pre-session positive affect and higher levels of negative affect; and (3) these symptom domains would moderate affective changes, such that individuals with lower baseline symptom levels would experience larger improvements in affect following the session. All hypotheses and analyses were preregistered at the Open Science Framework (OSF, https://osf.io/knc5r).

## Methods

### Participants

Eighty-two adult outpatients were recruited from 2 clinics in Vestfold Hospital Trust in Norway. Inclusion criteria were: (1) a Diagnostic and Statistical Manual of Mental Disorders (DSM) diagnosis of schizophrenia, confirmed using the Structured Clinical Interview for DSM-IV Axis I Disorders; (2) age 18-67 years; and (3) fluency in a Scandinavian language. Exclusion criteria included comorbid intellectual disability, pregnancy, or any medical condition contraindicating physical exercise. All participants provided written informed consent. Sixty-nine of the 82 randomized participants completed both pre- and post-session Positive and Negative Affect Schedule (PANAS) assessments at session 12 and were included in the preregistered analyses. Of these, 67 (97%) were receiving antipsychotic medication. The study was approved by the Regional Committee for Medical and Health Research Ethics (Project No. 2014/372).

### Study Design and Procedure

This study was part of the Effects of Physical Activity in Psychosis (EPHAPS) project (ClinicalTrials.gov, registration number NCT02205684). Detailed descriptions of the EPHAPS protocol, methodology, and sample size calculation have been reported previously.^10^^,^^25–27^ In summary, this was a randomized, observer-blinded, parallel-group controlled trial with a 1:1 allocation ratio. The original protocol was fully adhered to, except for an extended recruitment period to reach target sample size. Participants were randomly assigned, using a computer-generated allocation sequence, to a 12-week intervention consisting of either high-intensity interval training (HIIT) or an ACC, with 2 supervised sessions per week (~45 min per session). All outcome measurements except for sports physiological testing were assessor-blinded. Staff were present to ensure participant safety and engagement.

### Interventions

#### High-Intensity Interval Training (HIIT)

Participants in the HIIT group completed treadmill-based walking or running sessions. Sessions were conducted with 2 participants exercising simultaneously on individual treadmills under supervision by 2 staff members. Each session began with an 8-min warm-up, followed by four 4-min intervals at 85%-95% of the individual’s maximal heart rate (HRmax), separated by 3-min active recovery periods at 70% HRmax. Sessions ended with a 5-min cool-down. Target intensities were determined based on each participant’s measured HRmax during the baseline VO₂max test. Heart rate was continuously monitored with a Polar RCX3 chest-strap device to ensure adherence to the prescribed intensities during sessions.

#### Active Control Condition

Participants in the ACC engaged in low-intensity, interactive video gaming using Nintendo Wii Sport (tennis). Sessions were also conducted with 2 participants under supervision by 2 staff members. These sessions typically involved light physical activity, characterized by minor increases in heart rate and bodily movement, primarily requiring coordination rather than aerobic effort. The ACC was designed to account for time, attention, and social contact without producing substantial cardiovascular strain.

### Measurements

#### Affective State

Affective state was assessed using the PANAS.^28^ The self-report inventory comprises 20 items forming 2 subscales: a 10-item subscale for positive affect (e.g., enthusiastic, proud) and a 10-item subscale for negative affect (e.g., irritable, ashamed). Participants rated how they felt “right now” on a 5-point Likert scale (1 = very slightly or not at all; 5 = extremely). Each subscale yields a total score ranging from 10 to 50, with higher scores reflecting higher levels of the corresponding affect. The PANAS has demonstrated strong psychometric properties across non-clinical and clinical samples,^28–30^ including individuals with psychotic disorders in Scandinavian settings.^23^ The scale shows a stable 2-factor structure with high internal consistency (α ≈ 0.80-0.90) and is sensitive to momentary fluctuations in affect when assessing current affective state.^28^ The PANAS was administered 2-5 min before and after session 12 (HIIT or ACC). This session corresponded to the midpoint of the 12-week intervention (24 sessions total). In the HIIT condition, post-session assessment occurred approximately 7-10 min after the final high-intensity interval, following a standardized 5-min cool-down.

#### Apathy

Apathy was assessed with the Apathy Evaluation Scale, self-report version (AES^31^), an 18-item measure evaluating motivational and behavioral aspects of apathy (total score range: 18-72).

#### Depression

Depressive symptoms were assessed using the Calgary Depression Scale for Schizophrenia (CDSS^32^), a 9-item semi-structured interview designed to capture core depressive features in schizophrenia (total score range: 0-27).

#### Negative Symptoms

Negative symptoms were measured with the negative symptoms subscale of the Positive and Negative Syndrome Scale (PANSS^33^), a 7-item clinician-rated scale assessing blunted affect, emotional withdrawal, and related features (total score range: 7-49).

Unless stated otherwise, measures were collected at baseline (prior to the beginning of the 12-week intervention).

### Statistical Analysis

Changes in positive affect and negative affect were examined using linear mixed-effects models with fixed effects for Group (HIIT vs. ACC), Time (pre vs. post session), and their interaction, and a random intercept for participants. The confirmatory test of Hypothesis 1 was the Group × Time interaction. To test Hypothesis 2, we examined the association between baseline AES, CDSS, and PANSS-Negative total scores and pre-session positive affect and negative affect using linear regression models, with group entered as a covariate to control for potential differences between HIIT and ACC. Hypothesis 3 was tested by extending the mixed-effects models with 3-way interaction terms (Group × Time × Symptoms), with lower-order terms included. Each symptom variable (AES, CDSS, PANSS-Negative) was examined separately as a moderator, and scores were grand-mean centered. Measurement scores were set to missing if more than 20% of items were missing on a subscale. All analyses were conducted in R (version 4.4.0) using the lme4, lmerTest, and emmeans packages for mixed-effects modeling and estimated marginal means. Visualization was performed using ggplot2.

## Results

Demographic and clinical characteristics are presented in Table 1. Participants had a mean age of 38.4 years (*SD* = 14.6), and 61% were men. Duration of current illness averaged 17.6 years (SD = 13.9). Symptom severity was moderate, with mean PANSS total scores of 65.4 (*SD* = 17.13). Across interventions, negative symptoms (*M* = 18.2, *SD* = 7.3) were slightly more prominent than positive symptoms (*M* = 14.8, *SD* = 5.0). Depressive symptoms were low on average (CDSS: *M* = 3.1, *SD* = 3.6), and apathy scores indicated moderate levels of motivational impairment (AES: *M* = 39.2, *SD* = 9.0). The 2 groups did not differ significantly on any measured variable at baseline.

**Table 1:** Baseline Demographic Features and Clinical Scores of Participants

	Interventions		Comparison	
Characteristics	HIIT	ACC	Test statistic	*P*-value
N	32	37		
Gender, M	20	22	*x^2^* = 0.067	.796
Age (years)	37.2 (14.9)	39.5 (14.3)	*U* = 557.0	.857
Education (years)	12.4 (2.3)	12.1 (2.8)	t = 0.133	.693
Duration of illness (years)	16.2 (13.0)	16.0 (13.8)	*U* = 554.0	.810
VO₂max	30.17 (12.00)	29.48 (11.08)	*t* = 0.25	.805
AES	39.2 (9.7)	39.1 (8.2)	*t* = 0.204	.841
CDSS	2.7 (3.1)	3.4 (2.0)	*U* = 526.5	.857
PANAS negative affect	15.4 (6.4)	16.5 (6.9)	*U* = 584.0	.923
PANAS positive affect	26.8 (9.4)	26.1 (8.1)	*t* = −0.010	.992
PANSS total score	66.9 (17.9)	63.0 (16.5)	*t* = 1.47	.146
PANSS negative symptoms	19.0 (7.0)	17.1 (6.6)	*U* = 458.5	.223
PANSS positive symptoms	15.7 (5.1)	13.9 (4.8)	*t* = 1.347	.183

Values are means with standard deviations in parentheses unless otherwise specified. Normality of distributions was assessed using the Shapiro–Wilk test. Variables meeting assumptions of normality (*P* ≥ .05) were compared between groups using independent-samples t tests, whereas non-normally distributed variables (*P* < .05) were compared using Mann–Whitney U tests. Categorical variables were analyzed using *χ^2^* tests. Abbreviations: ACC = active control condition; AES = Apathy Evaluation Scale; CDSS = Calgary Depression Scale for Schizophrenia; HIIT = high-intensity interval training; PANAS = Positive and Negative Affect Schedule; PANSS = Positive and Negative Syndrome Scale.

**Figure 1:**
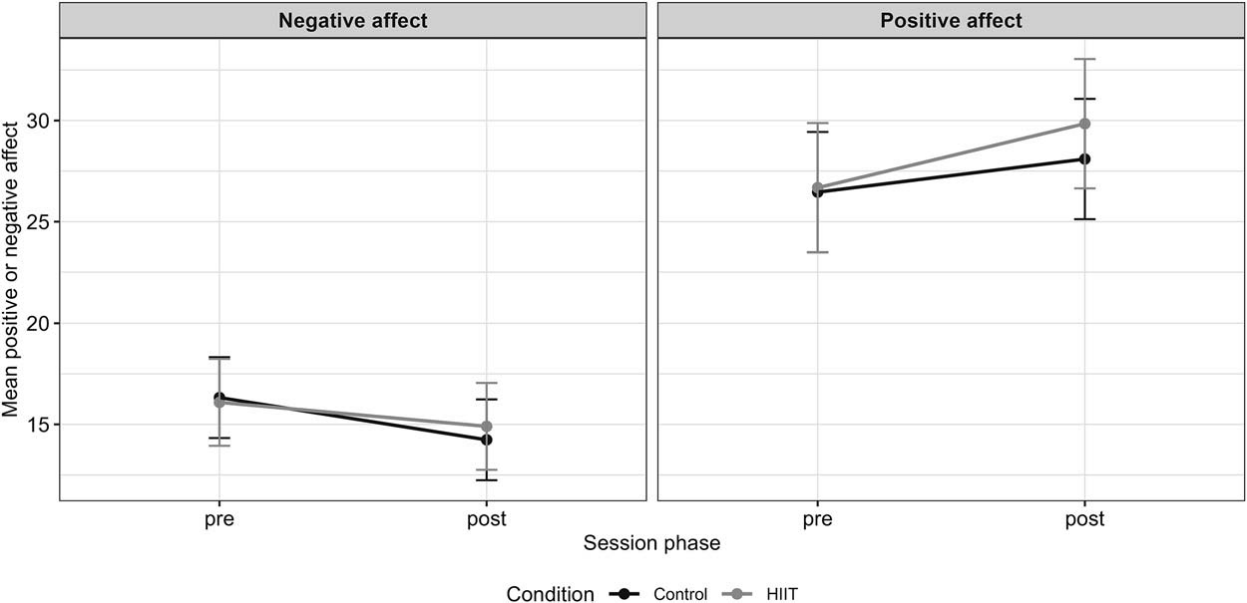
Estimated marginal means of positive and negative affect across session phases.

Contrary to expectations, the interaction term was not significant for either affect subscale (see Table 2). For positive affect, the additional HIIT-over-control change was *B* = 1.53, 95% CI, −0.90 to 3.96, *P* = .22. For negative affect, the additional HIIT-over-control change was *B* = 0.90, 95% CI, −0.94 to 2.75, *P* = .34. These results indicate no statistically significant advantage of HIIT over the ACC in eliciting affective change. Both groups showed improvements from pre- to post-session (see Figure 1 for visualization). There was a statistically significant increase in positive affect in the HIIT group, and no statistically significant change in the ACC. Negative affect did not change significantly in the HIIT group, and a significant decrease was observed in the ACC.

**Table 2:** Pre- to Post-Session Changes in Positive and Negative Affect Following HIIT Versus Active Control Condition

Variables	Positive affect			Negative affect		
**Between-group Δ**	***B***	**95% CI**	***p***	***B***	**95% CI**	***P***
HIIT × time	1.53	−0.90 to 3.96	.221	0.90	−0.94 to 2.75	.342
**Within-group Δ**	**Δ*M***	**95% CI**	***p***	**Δ*M***	**95% CI**	***P***
HIIT (Post–Pre)	3.17	1.36-4.98	**.001**	−1.19	−2.56 to 0.19	.090
ACC (Post–Pre)	1.64	−0.05 to 3.32	.057	−2.09	−3.37 to −0.81	**.002**

Between-group Δ represents unstandardized coefficients (*B*) from the Group × Time interaction in the mixed-effects model. Within-group Δ represents model-based estimated marginal mean differences (Δ*M*) from pre to post session. *P* = 2-tailed probability value. Boldface indicates *P* < .05. Abbreviations: ACC = active control condition; HIIT = high-intensity interval training. The Positive and Negative Affect Schedule (PANAS) was used to measure positive and negative affect.

To explore individual differences in affective state prior to activity engagement, we examined whether baseline symptom levels were associated with pre-session affect scores (see Table 3). Higher apathy scores (*B* = −0.53, *P* < .001), higher depressive symptoms (*B* = −0.85, *P* < .001), and higher negative symptom severity (*B* = −0.50, *P* = .002) were each significantly associated with lower pre-session positive affect, after adjusting for group. In contrast, none of the symptom measures were independently associated with pre-session negative affect (*P*s ≥ .14).

**Table 3:** Associations Between Baseline Symptom Levels and Pre-session Positive and Negative Affect

	Positive affect				Negative affect			
Variable	*B*	*SE*	*t*	*P*	*B*	*SE*	*t*	*P*
AES	−0.53	0.11	−4.89	**<.001**	−0.02	0.09	−0.23	.817
CDSS	−0.85	0.29	−2.97	**<.001**	0.31	0.21	1.48	.143
PANSS Negative	−0.50	0.16	−3.24	**.002**	−0.12	0.12	−0.98	.331

Unstandardized coefficients from separate linear regression models are reported. Boldface indicates *P* < .05. Abbreviations: AES = Apathy Evaluation Scale; CDSS = Calgary Depression Scale for Schizophrenia; PANSS = Positive and Negative Syndrome Scale. The Positive and Negative Affect Schedule (PANAS) was used to measure positive and negative affect.

Among the 6 moderation models, only the interaction between PANSS-Negative and Group × Time was statistically significant for positive affect (*P* = .04, see Table 4). No other 3-way interaction was statistically significant (*p*≥.36). For negative affect, none of the symptom variables moderated affective change (*p*≥.13). Given the exploratory nature of these analyses and the modest sample size, this interaction should be interpreted with caution and viewed as hypothesis-generating.

**Table 4:** Moderation of Pre-to-Post Affect Change by Baseline Symptom Levels

	Positive affect			Negative affect		
Moderator	*B*	95% CI	*P*	*B*	95% CI	*P*
Group x Time x AES	0.13	−0.16 to 0.42	.368	−0.17	−0.39 to 0.05	.132
Group x Time x CDSS	0.23	−0.46 to 0.91	.510	0.08	−0.42 to 0.58	.748
Group x Time x PANSS Negative	**0.38**	0.02 to 0.73	**.040**	−0.22	−0.47 to 0.04	.096

Coefficients represents the additional HIIT-over-control pre-to-post change in affect associated with a 1-point increase in the baseline symptom measure (3-way Group × Time × Symptom interaction). Positive coefficients indicate that the HIIT advantage grows with higher symptom severity; negative coefficients indicate the opposite. Boldface denotes *P* < .05 (exploratory, unadjusted). Abbreviations: AES = Apathy Evaluation Scale; CDSS = Calgary Depression Scale for Schizophrenia; PANSS = Positive and Negative Syndrome Scale. The Positive and Negative Affect Schedule (PANAS) was used to measure positive and negative affect.

## Discussion

HIIT provides a suitable context for investigating shifts in affective states in patients with schizophrenia. In this study of HIIT and an ACC, both activities elicited increases in positive affect and reductions in negative affect, with no significant between-group differences. Depressive symptoms, negative symptoms, and apathy were each independently associated with lower pre-session positive affect, and exploratory analyses suggested that individuals with more severe negative symptoms may experience somewhat greater affective gains from high-intensity training.

The absence of a significant difference between high- and low-effort physical activity suggests that short-term affective responses in people with schizophrenia may be less sensitive to exercise intensity than assumed. A plausible alternative explanation for the similar affective improvements across interventions is that both interventions shared features that may promote affective engagement, including structured activity, social contact, and behavioral activation. Consistent with this idea, people with schizophrenia have been found to report greater positive affect in social contexts despite often preferring solitude,^34^ suggesting that structured social environments may facilitate affective engagement. Both interventions may also have provided strong contextual scaffolding, characterized by clear guidance, predictable demands, and reduced initiation burden, that may have diminished the relative influence of exercise intensity.

Nonetheless, both interventions were associated with moderate improvements in affect, aligning with evidence indicating that structured and engaging activity can enhance affective experience.^18^^,^^19^ The results also align with evidence that individuals with schizophrenia retain intact consummatory pleasure in response to affectively engaging stimuli,^4–6^ and that affective experience varies meaningfully across situational contexts.^7–9^ Consistent with this, research shows that individuals with schizophrenia are capable of experiencing positive emotions in enjoyable situations, but encounter such contexts less frequently in daily life.^9^

Although evidence from non-clinical and other clinical populations is mixed and often limited by methodological constraints, many studies report increased positive affect or pleasure following moderate- or high-intensity training.^17^^,^^18^^,^^21^^,^^22^ This pattern is also consistent with evidence from a schizophrenia sample showing that a single bout of high-intensity training can increase positive affect.^19^ Evidence from other populations indicates that affective responses to exercise may affect subsequent exercise motivation,^35^^,^^36^ suggesting that even brief affective improvements may hold relevance for future engagement. These findings further support recent recommendations to consider affective response to exercise on treatment outcomes.^16^

Beyond group-level effects, individual differences in baseline symptom levels provided additional insights. As hypothesized, apathy, depressive symptoms, and negative symptoms, were each independently associated with pre-session positive affect. These findings indicate that motivational and mood-related symptom domains, while distinct from core affective capacity,^1^^,^^6^^,^^24^ still influence the affective state from which individuals engage with their environment. However, it is important to note that these symptom ratings were collected approximately 6 weeks prior to the session in which the PANAS scores were assessed. While symptom domains such as depression and negative symptoms are considered relatively stable over time,^37^^,^^38^ the temporal gap may have introduced measurement noise. Interestingly, the associations between baseline symptoms and pre-session affect were specific to positive affect.

Negative affect showed no significant relationship with baseline symptom levels. It was also unexpected that the ACC showed a significant within-group reduction in negative affect, whereas the HIIT group did not show a statistically significant change. The ACC may have offered a low-demand, predictable, and social activity more effective to negative affect, whereas high-intensity training may not down-regulate negative affect in the same manner. The absence of symptom-related associations with negative affect suggests that negative affect may be predominantly state-dependent and context-sensitive. In contrast, positive affect appears more closely tied to trait-like symptom domains such as negative symptoms. These patterns aligns with affective models in which positive affect and negative affect reflect independent systems.^28^^,^^30^

Our findings may also be understood in the context of affective trait models. Schizophrenia is characterized by relatively stable temperamental tendencies toward reduced enthusiasm, engagement, and pleasure (low trait positive affectivity) and heightened tendencies toward distress, worry, and sadness (high trait negative affectivity).^1^ Although the present study did not measure affective traits, such trait-level dispositions may help explain individual differences in the affective state with which participants entered the session. At the same time, the post-session affective changes observed in our data align with evidence that state-level affective reactivity remains largely intact despite these trait-level disturbances.^4–6^ This distinction between more stable affective traits and preserved momentary reactivity may help clarify how individuals can benefit from structured, engaging activities even when starting from a relatively subdued affective state.

Exploratory moderation analyses suggested that individuals with higher negative symptom severity might experience greater increases in positive affect following HIIT. Although this interaction should be interpreted cautiously due to limited power and lack of correction for multiple testing, it raises the possibility that some individuals with pronounced negative symptoms retain responsiveness to structured, high-intensity stimulation. This possibility is clinically relevant given the limited efficacy of pharmacological treatments for negative symptoms.^39–41^

### Clinical Implications

From a clinical perspective, the present findings suggest that both high-intensity training and socially structured activities may evoke beneficial affective shifts in individuals with schizophrenia. Interventions that capitalize on these affective shifts may thus help sustain motivation and adherence in long-term programs. These findings also have implications for interventions targeting the well-documented somatic health disparities in this population. People with schizophrenia experience elevated rates of cardiometabolic disorders and reduced life expectancy, partially which is attributable to a sedentary lifestyle.^42–44^ Even brief sessions of physical activity may potentially enhance both affective well-being and cardiometabolic health, and integrating affective monitoring could help identify intervention formats that optimize physiological benefits while also reinforcing engagement.

### Strengths and Limitations

The present study has several methodological strengths. It employed a randomized, observer-blinded, parallel-group design with an ACC, allowing a rigorous comparison of affective response to high-intensity interval training versus socially engaging video gaming. Both interventions were delivered according to supervised protocols, ensuring comparable structure. The preregistration of hypotheses and analytic procedures enhances transparency and reduces the risk of selective reporting.^45^ Together, these features strengthen the reproducibility of the findings. Baseline positive and negative affect scores in our sample closely resembled those reported in other Scandinavian schizophrenia cohorts,^19^^,^^23^ supporting the representativeness of our sample.

Several limitations warrant consideration. First, although the sample size is relatively large for an exercise RCT in schizophrenia, it may still have limited power to detect small effects, particularly for higher-order interactions. Second, affect was assessed using self-report only. While the PANAS is well-validated and widely used,^46^^,^^47^ it has been variously described in the literature as a measure of mood, emotion, or affective state,^46^ reflecting conceptual ambiguity in the field. Caution is therefore warranted when generalizing our findings across distinct affective constructs. Future studies could incorporate additional measures of affect, including physiological, neurological, or behavioral indicators. Third, affect was assessed shortly after session completion, capturing immediate affective responses but not later changes that may occur following high-intensity exercise.^21^^,^^22^ Forth, our single-session design limits generalizability to repeated or long-term exercise exposure. Future research should examine whether affective changes accumulate across sessions or predict adherence and functional outcomes. Fifth, physiological and contextual factors such as perceived exertion, cardiovascular response were not assessed. In addition, potential differences between conditions in how participants interacted socially with staff or with each other were not measured, which may have influenced the contrast between groups in affective change. Finally, the sample consisted of clinically stable outpatients with schizophrenia, which limits generalizability to patients with more pronounced symptomatology.

### Conclusion

In this randomized controlled trial, both high-intensity interval training and socially engaging video-based activity produced improvements in affect among individuals with schizophrenia. Our findings suggest that structured, affectively engaging interventions, regardless of physical intensity, may yield psychological benefits. Apathy, depressive symptoms, and negative symptoms were associated with lower pre-session positive affect, while exploratory findings indicated that individuals with more severe negative symptoms may benefit most from high intensity training. Future research should investigate how brief affective improvements translate into sustained affective well-being, adherence, and somatic health gains.

## Notes

Authors starting at the 3rd author position and except for the last author are alphabetically ordered.
